# Supplementary material for: Surgical Trends and Complications in Partial and Radical Nephrectomy: Results from the GRAND Study
Source: Cancers (Basel). 2023 Dec 24;16(1):97. doi: 10.3390/cancers16010097 (PMC10778168; doi:10.3390/cancers16010097)
Supplement: Supplementary file 1 [file cancers-16-00097-s001.zip › cancers-2788290-supplementary.pdf]

# Supplementary Material

## Table of Contents


***Supplementary Material Table S1: Baseline characteristics in low-, intermediate- and high-volume centers performing renal surgeries ........ 2***

***Supplementary Material Figure S1: Annual trends in low-, intermediate- and high-volume centers performing renal surgeries ........ 5***

***Supplementary Material Table S2: Regression analysis in low-, intermediate- and high-volume centers performing renal surgeries ........ 6***

***Supplementary Material Figure S2: 30-day survival in low-, intermediate- and high-volume centers performing renal surgeries ........ 8***

## Supplementary Material Table S1: Baseline characteristics in low-, intermediate- and high-volume centers performing renal surgeries.

| **Characteristic** | **Overall**, n 1 57,308 | **Partial nephrectomy**, n = 17,108 | **Radical nephrectomy**, n = 40,200 | **p-value** |
|---|---|---|---|---|
| **Age (years)** | 68 (58-76) | 68 (57-74) | 68 (58-76) | **<0.001** |
| **Males** | 32,794 (57%) | 10,391 (61%) | 22,403 (56%) | **<0.001** |
| **Diabetes** | 10,933 (19%) | 3,172 (19%) | 7,761 (19%) | **0.034** |
| **Chronic heart failure** | 4,856 (8.5%) | 1,171 (6.8%) | 3,685 (9.2%) | **<0.001** |
| **Chronic obstructive pulmonary disease** | 4,569 (8.0%) | 1,342 (7.8%) | 3,227 (8.0%) | 0.47 |
| **Chronic kidney disease** | 11,743 (20%) | 2,433 (14%) | 9,310 (23%) | **<0.001** |
| **Cerebrovascular disease** | 1,593 (2.8%) | 341 (2.0%) | 1,252 (3.1%) | **<0.001** |
| **Dementia** | 941 (1.6%) | 143 (0.8%) | 798 (2.0%) | **<0.001** |
| **Hypertension** | 33,546 (59%) | 10,186 (60%) | 23,360 (58%) | **0.002** |
| **Obesity** | 6,159 (11%) | 1,884 (11%) | 4,275 (11%) | 0.19 |
| **Operative technique** | | | | **<0.001** |
| Open | 51,464 (90%) | 14,903 (87%) | 36,561 (91%) | |
| Laparoscopic | 4,925 (8.6%) | 1,528 (8.9%) | 3,397 (8.5%) | |
| Robotic | 919 (1.6%) | 677 (4.0%) | 242 (0.6%) | |

Supplementary Material Table S1A: Baseline characteristics of the included patients based on the type of renal cancer surgery in low-volume centers. Variables are presented as median with interquartile range or frequencies with proportions. The Mann-Whitney test was performed for comparisons between continuous variables and the chi-squared test for categorical variables. The bold cells indicate statistically significant p-values.

| **Characteristic** | **Overall**, n = 142,702 | **Partial nephrectomy**, n = 52,966 | **Radical nephrectomy**, n = 89,736 | **p-value** |
|---|---|---|---|---|
| **Age (years)** | 67 (57-75) | 66 (57-74) | 68 (57-76) | **<0.001** |
| **Males** | 83,733 (59%) | 32,700 (62%) | 51,033 (57%) | **<0.001** |
| **Diabetes** | 26,696 (19%) | 9,683 (18%) | 17,013 (19%) | **0.002** |
| **Chronic heart failure** | 9,276 (6.5%) | 2,548 (4.8%) | 6,728 (7.5%) | **<0.001** |
| **Chronic obstructive pulmonary disease** | 10,817 (7.6%) | 3,895 (7.4%) | 6,922 (7.7%) | **0.013** |
| **Chronic kidney disease** | 29,433 (21%) | 7,369 (14%) | 22,064 (25%) | **<0.001** |
| **Cerebrovascular disease** | 3,620 (2.5%) | 1,009 (1.9%) | 2,611 (2.9%) | **<0.001** |
| **Dementia** | 1,879 (1.3%) | 343 (0.6%) | 1,536 (1.7%) | **<0.001** |
| **Hypertension** | 82,427 (58%) | 31,104 (59%) | 51,323 (57%) | **<0.001** |
| **Obesity** | 14,845 (10%) | 5,564 (11%) | 9,281 (10%) | 0.34 |
| **Operative technique** | | | | **<0.001** |
| Open | 113,349 (79%) | 39,619 (75%) | 73,730 (82%) | |
| Laparoscopic | 21,358 (15%) | 7,102 (13%) | 14,256 (16%) | |
| Robotic | 7,995 (5.6%) | 6,245 (12%) | 1,750 (2%) | |

Supplementary Material Table S1B: Baseline characteristics of the included patients based on the type of renal cancer surgery in intermediate-volume centers. Variables are presented as median with interquartile range or frequencies with proportions. The Mann-Whitney test was performed for comparisons between continuous variables and the chi-squared test for categorical variables. The bold cells indicate statistically significant p-values

| **Characteristic** | **Overall**, n = 117,833 | **Partial nephrectomy**, n = 53,850 | **Radical nephrectomy**, n = 63,983 | **p-value** |
|---|---|---|---|---|
| **Age (years)** | 65 (54-73) | 64 (55-72) | 65 (54-73) | **0.009** |
| **Males** | 71,596 (61%) | 34,026 (63%) | 37,570 (59%) | **<0.001** |
| **Diabetes** | 19,526 (17%) | 8,464 (16%) | 11,062 (17%) | **<0.001** |
| **Chronic heart failure** | 6,008 (5.1%) | 2,003 (3.7%) | 4,005 (6.3%) | **<0.001** |
| **Chronic obstructive pulmonary disease** | 7,756 (6.6%) | 3,457 (6.4%) | 4,299 (6.7%) | **0.04** |
| **Chronic kidney disease** | 24,429 (21%) | 6,578 (12%) | 17,851 (28%) | **<0.001** |
| **Cerebrovascular disease** | 2,394 (2.0%) | 776 (1.4%) | 1,618 (2.5%) | **<0.001** |
| **Dementia** | 869 (0.7%) | 207 (0.4%) | 662 (1.0%) | **<0.001** |
| **Hypertension** | 63,413 (54%) | 29,092 (54%) | 34,321 (54%) | 0.19 |
| **Obesity** | 11,004 (9.3%) | 4,936 (9.2%) | 6,068 (9.5%) | 0.06 |
| **Operative technique** | | | | **<0.001** |
| Open | 84,520 (72%) | 34,705 (64%) | 49,815 (78%) | |
| Laparoscopic | 18,711 (16%) | 6,898 (13%) | 11,813 (18%) | |
| Robotic | 14,602 (12%) | 12,247 (23%) | 2,355 (3.7%) | |

Supplementary Material Table S1C: Baseline characteristics of the included patients based on the type of renal cancer surgery in high-volume centers. Variables are presented as median with interquartile range or frequencies with proportions. The Mann-Whitney test was performed for comparisons between continuous variables and the chi-squared test for categorical variables. The bold cells indicate statistically significant p-values.

# Supplementary Material Figure S1: Annual trends in low-, intermediate- and high-volume centers performing renal surgeries

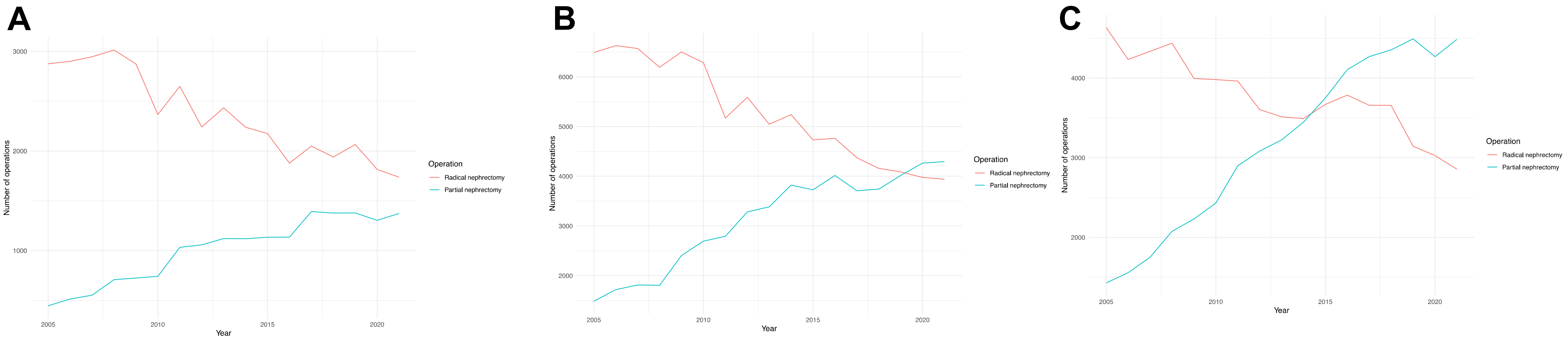


Supplementary Material Figure S1: The annual trends for radical and partial nephrectomy in low- (2A), intermediate- (2B), and high-volume centers (2C).

# Supplementary Material Table S2: Regression analysis in low-, intermediate- and high-volume centers performing renal surgeries.

| | | Transfusion | | | Sepsis | | | Acute respiratory failure | | | Acute kidney disease | | | Acute thromboembolism | | | Surgical wound infection | | |
|---|---|---|---|---|---|---|---|---|---|---|---|---|---|---|---|---|---|---|---|
| **Annual caseload** | **Surgery** | **Events** | **OR** | **p-value** | **Events** | **OR** | **p-value** | **Events** | **OR** | **p-value** | **Events** | **OR** | **p-value** | **Events** | **OR** | **p-value** | **Events** | **OR** | **p-value** |
| Low-volume centers (<40) | Partial nephrectomy | 2,487 (15%) | — | — | 226 (1.3%) | — | — | 969 (5.7%) | — | — | 768 (4.5%) | — | — | 76 (0.4%) | — | — | 82 (0.5%) | — | — |
| | Radical nephrectomy | 10,243 (25%) | 1.7 (1.6, 1.8) | **<0.001** | 1,274 (3.2%) | 2.2 (1.9, 2.56) | **<0.001** | 2,595 (6.5%) | 1.3 (1.2, 1.4) | **<0.001** | 2,397 (6%) | 1.6 (1.5, 1.7) | **<0.001** | 303 (0.8%) | 1.7 (1.3, 2.2) | **<0.001** | 328 (0.8%) | 1.5 (1.2, 2) | **<0.001** |
| Intermediate-volume centers (40-99) | Partial nephrectomy | 6,375 (12%) | — | — | 550 (1%) | — | — | 2,136 (4%) | — | — | 2,293 (4.3%) | — | — | 219 (0.4%) | — | — | 188 (0.4%) | — | — |
| | Radical nephrectomy | 21,232 (24%) | 1.9 (1.9, 2) | **<0.001** | 2,675 (3%) | 2.5 (2.3, 2.8) | **<0.001** | 4,902 (5.5%) | 1.5 (1.4, 1.6) | **<0.001** | 5,376 (6.0%) | 1.6 (1.5, 1.7) | **<0.001** | 711 (0.8%) | 2 (1.7, 2.3) | **<0.001** | 635 (0.7%) | 1.9 (1.6, 2.2) | **<0.001** |
| High-volume centers (≥100) | Partial nephrectomy | 6,453 (12%) | — | — | 484 (0.9%) | — | — | 1,343 (2.5%) | — | — | 1,924 (3.6%) | — | — | 335 (0.6%) | — | — | 121 (0.2%) | — | — |
| | Radical nephrectomy | 17,694 (28%) | 2.3 (2.2, 2.4) | **<0.001** | 2,069 (3.2%) | 2.9 (2.6, 3.2) | **<0.001** | 2,941 (4.6%) | 1.8 (1.7, 2) | **<0.001** | 3,635 (5.7%) | 1.6 (1.5, 1.7) | **<0.001** | 788 (1.2%) | 2 (1.8, 2.3) | **<0.001** | 406 (0.6%) | 2.4 (2, 3) | **<0.001** |

Supplementary Material Table S2A: Multivariable logistic regression analysis in low-, intermediate- and high-volume centers for partial and radical nephrectomy on transfusion, sepsis, acute respiratory failure, acute kidney disease, acute thromboembolism, and surgical wound infection. All models are adjusted for sex, age, obesity, history of chronic obstructive pulmonary disease, chronic heart failure, chronic kidney disease, cerebrovascular accident, hypertension, diabetes, surgical approach, and year of operation. The bold cells indicate statistically significant p-values. OR: odds ratio.

| | | Ileus | | | 30-day mortality | | | ICU admission | | | Length of hospital stay | | | Costs | | | Pancreatitis | | |
|---|---|---|---|---|---|---|---|---|---|---|---|---|---|---|---|---|---|---|---|
| **Annual caseload** | **Surgery** | **Events** | **OR** | **p-value** | **Events** | **Euros** | **Beta** | **Euros** | **Beta** | **Euros** | **Beta** | **Beta** | **p-value** | **Euros** | **Beta** | **p-value** | **Events** | **OR** | **p-value** |
| Low-volume centers (<40) | Partial nephrectomy | 317 (1.9%) | — | — | 116 (0.7%) | — | — | 3,398 (20%) | — | — | 10 (8-12) | — | — | 7,087 (6,332-8,408) | — | — | 39 (0.2%) | — | — |
| | Radical nephrectomy | 947 (2.4%) | 1.2 (1.1, 1.4) | **0,001** | 917 (2.3%) | 2.8 (2.3, 3.5) | **<0.001** | 8,899 (22%) | 1 (0.98, 1.1) | 0,2 | 11 (9-16) | 1.8 (1.6, 2) | **<0.001** | 7,311 (6,320-10,252) | 1,607 (1,400, 1,814) | **<0.001** | 147 (0.4%) | 1.6 (1.1, 2.3) | **0,013** |
| Intermediate-volume centers (40-99) | Partial nephrectomy | 709 (1.3%) | — | — | 234 (0.4%) | — | — | 9,348 (18%) | — | — | 9 (7, 11) | — | — | 7,087 (6,500-8,145) | — | — | 73 (0.1%) | — | — |
| | Radical nephrectomy | 1,725 (1.9%) | 1.4 (1.3, 1.5) | **<0.001** | 1,704 (1.9%) | 3.6 (3.1, 4.1) | **<0.001** | 19,537 (22%) | 1.1 (1, 1.1) | **<0.001** | 11 (8, 15) | 1.5 (1.4, 1.6) | **<0.001** | 7,314 (6,472-10,252) | 1,398 (1,289, 1,507) | **<0.001** | 222 (0.2%) | 1.6 (1.2, 2.1) | **0,001** |
| High-volume centers (≥100) | Partial nephrectomy | 597 (1.1%) | — | — | 190 (0.4%) | — | — | 7,370 (14%) | — | — | 8 (6, 10) | — | — | 7,087 (6,544-7,920) | — | — | 54 (0.1%) | — | — |
| | Radical nephrectomy | 1,231 (1.9%) | 1.4 (1.3, 1.6) | **<0.001** | 1,041 (1.6%) | 3.7 (3.2, 4.4) | **<0.001** | 12,919 (20%) | 1.5 (1.5, 1.6) | **<0.001** | 10 (7, 14) | 2.3 (2.2, 2.5) | **<0.001** | 7,664 (6,768-10,946) | 2,415 (2,261, 2,570) | **<0.001** | 267 (0.4%) | 3.4 (2.5, 4.6) | **<0.001** |

Supplementary Material Table S2B: Multivariable logistic regression analysis in low-, intermediate- and high-volume centers for partial and radical nephrectomy on ileus, 30-day mortality, ICU admission, length of hospital stay, costs, and pancreatitis. All models are adjusted for sex, age, obesity, history of chronic obstructive pulmonary disease, chronic heart failure, chronic kidney disease, cerebrovascular accident, hypertension, diabetes, surgical approach, and year of operation. The bold cells indicate statistically significant p-values. ICU: intensive care unit, OR: odds ratio.

## Supplementary Material Figure S2: 30-day survival in low-, intermediate- and high-volume centers performing renal surgeries.

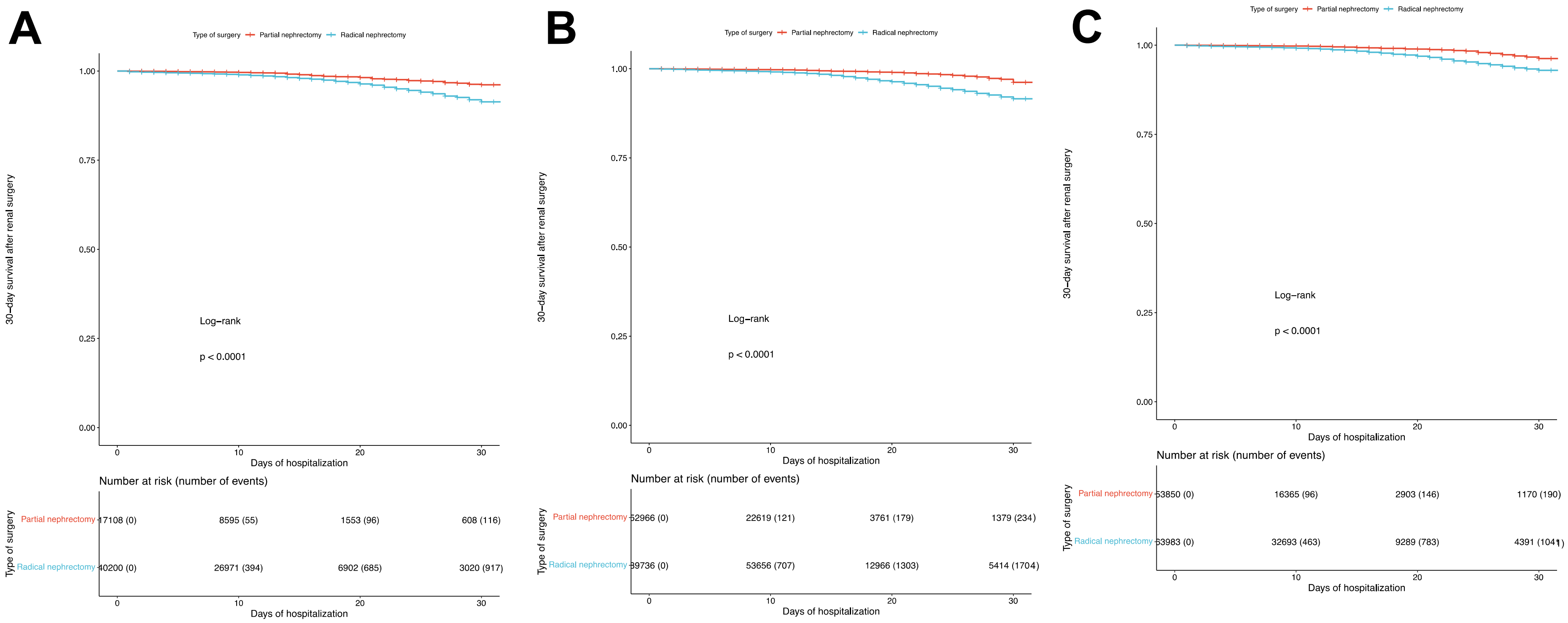


Supplementary Material Figure S2: Kaplan-Maier curve for the 30-day survival in patients undergoing radical versus partial nephrectomy in low- (4A), intermediate- (4B), and high-volume centers (4C).
